# Voiding defects in acute radiation cystitis driven by urothelial barrier defect through loss of E-cadherin, ZO-1 and Uroplakin III

**DOI:** 10.1038/s41598-021-98303-2

**Published:** 2021-09-29

**Authors:** Bernadette M. M. Zwaans, Alexander L. Carabulea, Sarah N. Bartolone, Elijah P. Ward, Michael B. Chancellor, Laura E. Lamb

**Affiliations:** 1 Beaumont Health System, Medical Office Building, 3535 W. 13 Mile Road, Suite 438, Royal Oak, MI 48073 USA; 2 Oakland University William Beaumont School of Medicine, Rochester, MI USA

**Keywords:** Diseases, Medical research, Urology

## Abstract

Long term-side effects from cancer therapies are a growing health care concern as life expectancy among cancer survivors increases. Damage to the bladder is common in patients treated with radiation therapy for pelvic cancers and can result in radiation (hemorrhagic) cystitis (RC). The disease progression of RC consists of an acute and chronic phase, separated by a symptom-free period. Gaining insight in tissue changes associated with these phases is necessary to develop appropriate interventions. Using a mouse preclinical model, we have previously shown that fibrosis and vascular damage are the predominant pathological features of chronic RC. The goal of this study was to determine the pathological changes during acute RC. We identified that radiation treatment results in a temporary increase in micturition frequency and decrease in void volume 4–8 weeks after irradiation. Histologically, the micturition defect is associated with thinning of the urothelium, loss of urothelial cell–cell adhesion and tight junction proteins and decrease in uroplakin III expression. By 12 weeks, the urothelium had regenerated and micturition patterns were similar to littermate controls. No inflammation or fibrosis were detected in bladder tissues after irradiation. We conclude that functional bladder defects during acute RC are driven primarily by a urothelial defect.

## Introduction

Decades of extensive cancer research and subsequent improved cancer screenings and treatments have resulted in an increasing number of patients overcoming their cancer diagnosis. According to the American Cancer Society, 16.9 million cancer survivors were living in the United States as of January 2019^1^. This population is estimated to increase with 30% by 2030^1^. However, many cancer survivors suffer long-term side effects from their cancer therapies^1^. Another 1.9 million people are expected to be diagnosed with cancer in 2021, of which about two thirds will receive radiation therapy, either alone or in combination with other treatments^1^. Radiation treatment to the pelvis for pelvic cancers such as prostate, colorectal, ovarian, and cervical cancer can cause lasting damage to the urinary bladder that can develop into radiation cystitis (RC), also known as hemorrhagic cystitis when bleeding is present^2–5^. It is estimated that approximately 10% of cancer survivors who received pelvic radiation therapy will develop RC^6^.

RC has three distinct phases—the acute, latent, and chronic phases^2^. The acute phase occurs during and shortly after completion of pelvic radiation therapy. Patients experience lower urinary tract symptoms such as frequency, urgency, and nocturia. This typically lasts only a few weeks. The acute phase is followed by the asymptomatic latent phase, which can last months to years. While the patient does not have any symptoms during the latent phase, fibrotic tissue is gradually accumulating, and vascular remodeling is taking place. Eventually the patient enters the chronic phase, during which the patient has evident symptoms of bladder disease including frequency, urgency, nocturia, incontinence, dysuria, pelvic pain, and hematuria^2–5^. While each of these symptoms severely impacts the quality-of-life of a cancer survivor, the presence of hematuria can make RC a life-threatening condition thus arresting the bleeding becomes the focus of treatment^7^. Several treatment options are available, such as instillations with astringent agents, hyperbaric oxygen therapy, or, in the most severe cases, cystectomy. However, these treatments are time-consuming and can have their own severe side effects^8^. A significant number of patients will relapse and none of these therapies can reverse the tissue damage. After hyperbaric oxygen therapy, partial to full resolution of hematuria is estimated at 64–100%, with reported recurrence rates of hematuria up to 36% within the first 12 months^9^. Thus, there is dire need for improved therapies and means of early diagnosing patients.

The development of novel therapies and diagnostic tools for RC rely on understanding the radiation-induced changes to the bladder in each of the phases. We have previously developed a preclinical RC model that closely mimics the human condition^10^. In this model, we have shown that, during the chronic phase of RC, radiation exposure results in long-term micturition defects, including frequency and decreased bladder capacity. These bladder defects coincide with an accumulation of fibrotic tissue and vascular damage (e.g. hemorrhaging and telangiectasia)^10^. In support of this, we have identified increased pro-fibrotic and vascular health markers in the urine of prostate cancer survivors with RC^11,12^. To fully understand the bladder changes that characterize the different phases of RC, the goal of this study was to determine the mechanism that induces the symptoms during the acute phase of RC.

## Material and methods

### Animals

All experimental procedures were reviewed and approved by Beaumont Health’s Animal Care Committee (AL-16-02). All experiments were performed in accordance with IACUC guidelines and regulations, and authors complied with ARRIVE guidelines. Female C57BL/6 mice (Charles River), eight weeks at the onset of the study, were housed under standard housing conditions with 4–5 mice per cage, fed a soy protein-free extrudent rodent diet, received water through water bottles, and their cages were changed weekly. Female mice were chosen as they are anatomically easier to catheterize. The C57BL/6 mouse strain was chosen as we have previously shown that this strain is sensitive to radiation-induced bladder fibrosis, loss of bladder vascularization, and voiding defect^10^. Animal treatment and experiments are outlined in Supplemental Fig. S1.

### Radiation treatment

Radiation was performed on the SARRP unit (Xstrahl Life Sciences) as previously described^10,13^. In brief, eight-week-old female C57BL/6 mice were anesthetized using isoflurane, bladder was emptied through gentle expression, and animals were positioned on the SARRP mouse platform and maintained on 1.5–2.0% isoflurane throughout the procedure. A computerized tomography was taken and the target for irradiation set in the middle of the bladder. A 5 × 5 mm SARRP collimator was installed and a total dose of 40 Gray was delivered through two separate beams. Control animals were anesthetized for the same duration as irradiated mice, without receiving radiation treatment. Imaging and irradiation for each animal was accomplished within 30–60 min. Following successful irradiation, mice recovered in a warming tank and returned to general housing. Animals were given mash during the first week after radiation treatment to ensure easy access to food.

### Cyclophosphamide treatment

Cyclophosphamide (CYP) treatment was used as a positive control for bladder inflammation. CYP was diluted in 10 ml of saline and given to mice through an IP injection at a dose of 300 mg CYP/kg—for 25 g mouse 7.5 mg/250 ul was given^14^. Prior to CYP injection, a single dose of 1 mg/kg sustained release buprenex (Buprenex-SR) was given to prevent and treat pain. Bladders were harvested 24 h after injection for downstream protein analysis or histology.

### Micturition assay

Micturition measurements were performed as previously described with minor alterations^13^. Once per week, mice were placed into metabolic cages for overnight voiding measurements (16 h). During the assay, mice had access to food and hydrogel, which was used instead of water to avoid water spills. PH sensitive paper was affixed to the underside of the cage to collect urine spots. Urinary frequency was calculated by quantifying the number of urine spots on the paper, and urine output was estimated by measuring the surface area of the urine spots, comparing to a standard curve of known volume/area measurements using a Photoshop counting tool. The baseline time point consisted of the average of three micturition assays performed prior to irradiation treatment. The moving average of 3 consecutive measurements was calculated and plotted.

### Luminex assay

At time of sacrifice, bladders intended for protein analysis were dissected, frozen in liquid nitrogen and stored at − 80 °C. Each frozen bladder tissue was placed in a 1.5 ml microcentrifuge tube containing four 0.9–2 mm beads (Next Advance, SSB14B) and 100 µl lysis buffer (0.1% tween 20 in PBS with proteinase inhibitor cocktail (Thermo Fisher Scientific, A32955)). Tubes were placed in Bullet Blender Blue (Next Advance, BBx24B) and bladders were homogenized for 3 min at maximum speed. Samples were spun down and supernatant collected. Protein analysis was performed using the Milliplex Multiplex Assay System (Millipore, Billerica, MA) according to manufacturer’s instructions. All samples were run in duplicate and technical quality controls were included during the assay. Cytokine concentrations were normalized to total protein and expressed in pg/10 mg of total protein. Total protein concentration for each sample was measured using the Pierce BCA protein assay (Thermo Fisher Scientific).

### Immunohistochemistry

At time of sacrifice, bladders designated for histology were instilled with 100 µl of 4% formaldehyde, dissected, fixed for 24 h at 4 °C, and transferred to 70% EtOH. Subsequently, bladders were cut in half longitudinally, and processed for histology at the University of Michigan ULAM in-vivo animal core facility.

5 µm sections were subjected to H&E or Masson’s Trichrome (fibrosis; TRM-2, SCYTEK laboratories) staining. Additionally, slides were immunostained for E-cadherin (ab76055, Abcam),Uroplakin III (ab78196, Abcam) and ZO-1 (61-7300, Thermo Fisher Scientific). All slides were warmed to 60 °C for a minimum of 20 min and subjected to a series of deparaffinization and rehydration steps. Trichrome staining was performed according to the manufacturer’s instructions. For E-cadherin and uroplakin III, heat-induced antigen retrieval was performed in 10 mM sodium citrate buffer pH6.0 for 20 min. Staining was subsequently performed using M.O.M. kit (PK-2200, Vector Labs) according to the manufacturer’s instructions, with a 15–30 min incubation for primary antibodies. Antibody signals were visualized with Immpact DAB (Vector Labs, SK-4105) and counterstained with hematoxylin (Vector Labs, H-3404). For ZO-1, antigen retrieval was performed for 5 min with Proteinase K solution (S302080, Agilent). Slides were blocked in 10% normal goat serum in TBS with 0.01% tween for 1 h. Sections were incubated sequentially for 1 h at room temperature with primary antibody and goat anti-rabbit Alexa Fluor 488 (1:500; A11070, Thermo Fisher Scientific). Both antibodies were diluted in antibody diluent (1%BSA in TBS). Slides were mounted using ProLong Diamond Antifade Mountant (P36970, Thermo Fisher Scientific). All primary antibodies were diluted 1:100. Imaging was performed on Olympus BX43 microscope.

### Data analysis

All data points were processed in GraphPad and statistical significance was calculated using the multiple T-test. For micturition data, averages were calculated between mice per treatment and time point. Subsequently, the moving average was calculated over 3 consecutive time points and significant differences were determined using the multiple T-test. For Luminex assay, irradiated and control groups included bladders harvested between 48 h and 12 weeks after radiation treatment. Cytokine concentrations were normalized to total protein as described above. The average normalized level of cytokine per group was calculated. A two-way ANOVA was performed to determine statistical significant interaction between treatment and time-point on inflammatory cytokine expression. Two-way ANOVA was followed by Bonferroni post-hoc test to identify difference between untreated and irradiated bladders at each time point. T-test was used to identify statistically significant difference in expression in cytokines between CYP and CYP control bladders.

For Masson’s Trichrome staining, data analysis was performed as previously described^10^. In short, six representative images were taken per bladder tissue. The amount of fibrosis for each bladder was quantified using the color deconvolution tool in ImageJ. Per image, 3 regions of interest were used to quantify the percent of tissue positive for fibrosis (blue stain). The average percent of positive fibrosis staining was calculated for each bladder, and subsequently the averages were calculated per treatment group.

## Results

### Radiation exposure to the bladder results in a transient bladder voiding defect

**Figure 1:**
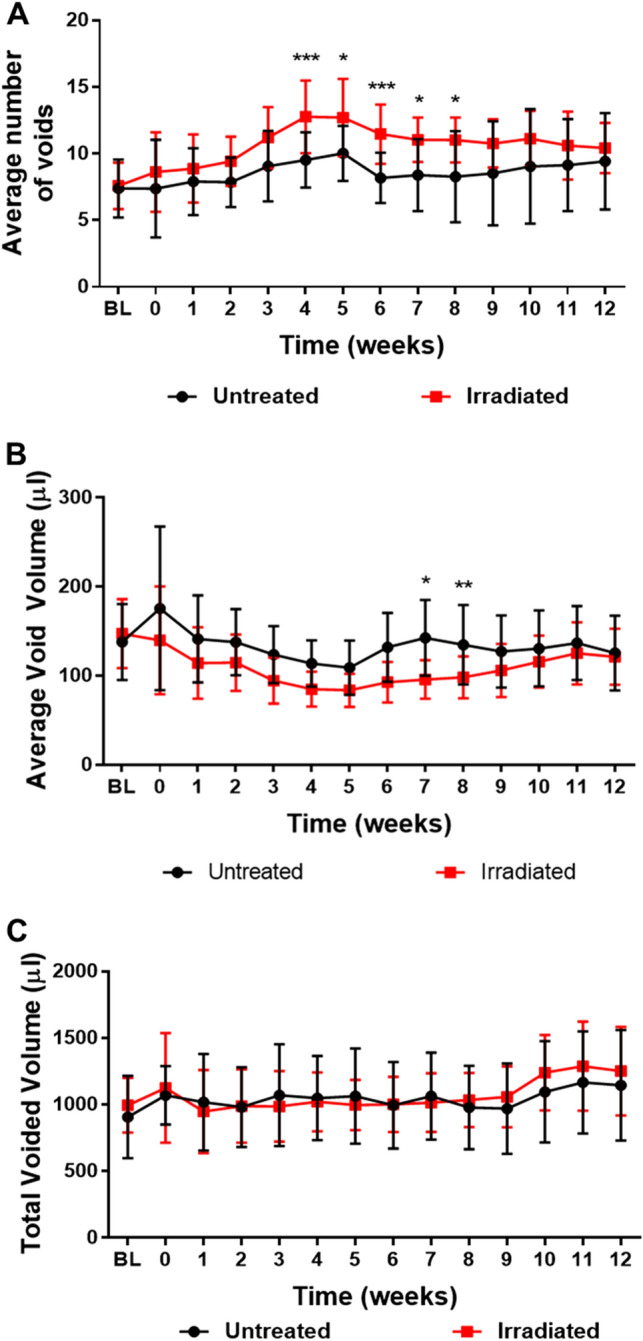
Increased frequency and reduced void volume due to irradiation exposure. (**A**) The average number of voids, (**B**) the average volume per void, (**C**) and the total voided volume were plotted for untreated and irradiated treatment groups from baseline (BL) to 12 weeks post-irradiation. *p < 0.05; **p < 0.01; ***p < 0.001. Error bars = SD.

Mice tolerated radiation treatment well as no change in body weight and behavior was detected compared to non-irradiated littermates (Suppl. Fig. S2). To assess the effect of radiation treatment on bladder function, mice were subjected to weekly micturition measurements. A significant increase in voiding frequency was observed in the mice that received radiation treatment between 4 and 8 weeks post-irradiation (Fig. 1A). Similarly, a significant decrease in the average volume per void was detected at 7 and 8 weeks after irradiation (Fig. 1B) while the total voided volume (Fig. 1C) remained unchanged. There was no difference in bladder function, as measured by micturition frequency and average void volume, between irradiated and control mice at 12 weeks post irradiation exposure.

### Urothelial thinning and permeability in irradiated bladder tissue coincides with voiding defect

**Figure 2:**
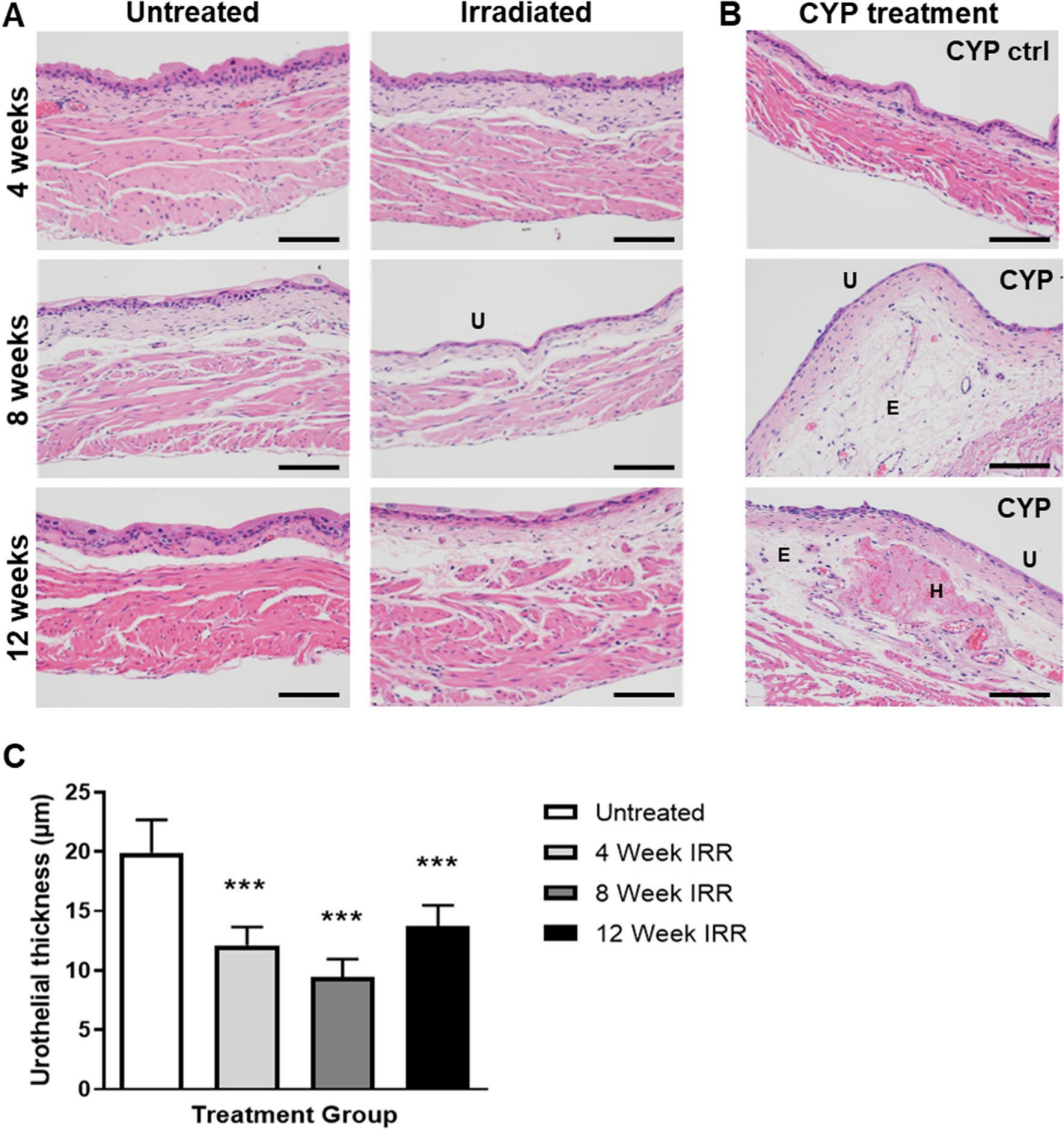
Irradiation induces temporary thinning of the bladder urothelium. (**A**) Untreated and irradiated bladder tissues were stained for Hematoxylin and Eosin at 4, 8 and 12 weeks after radiation treatment. (**B**) Bladders treated with cyclophosphamide (CYP), a positive control for inflammation, were stained with H&E 24 h after treatment. (**C**) Urothelial thickness was quantified for untreated bladders and 4, 8, and 12 weeks after irradiation. *U* urothelial thinning, *E* edema, *H* hemorrhaging; Scale bar = 100 µm; Error bar = SD.

**Figure 3:**
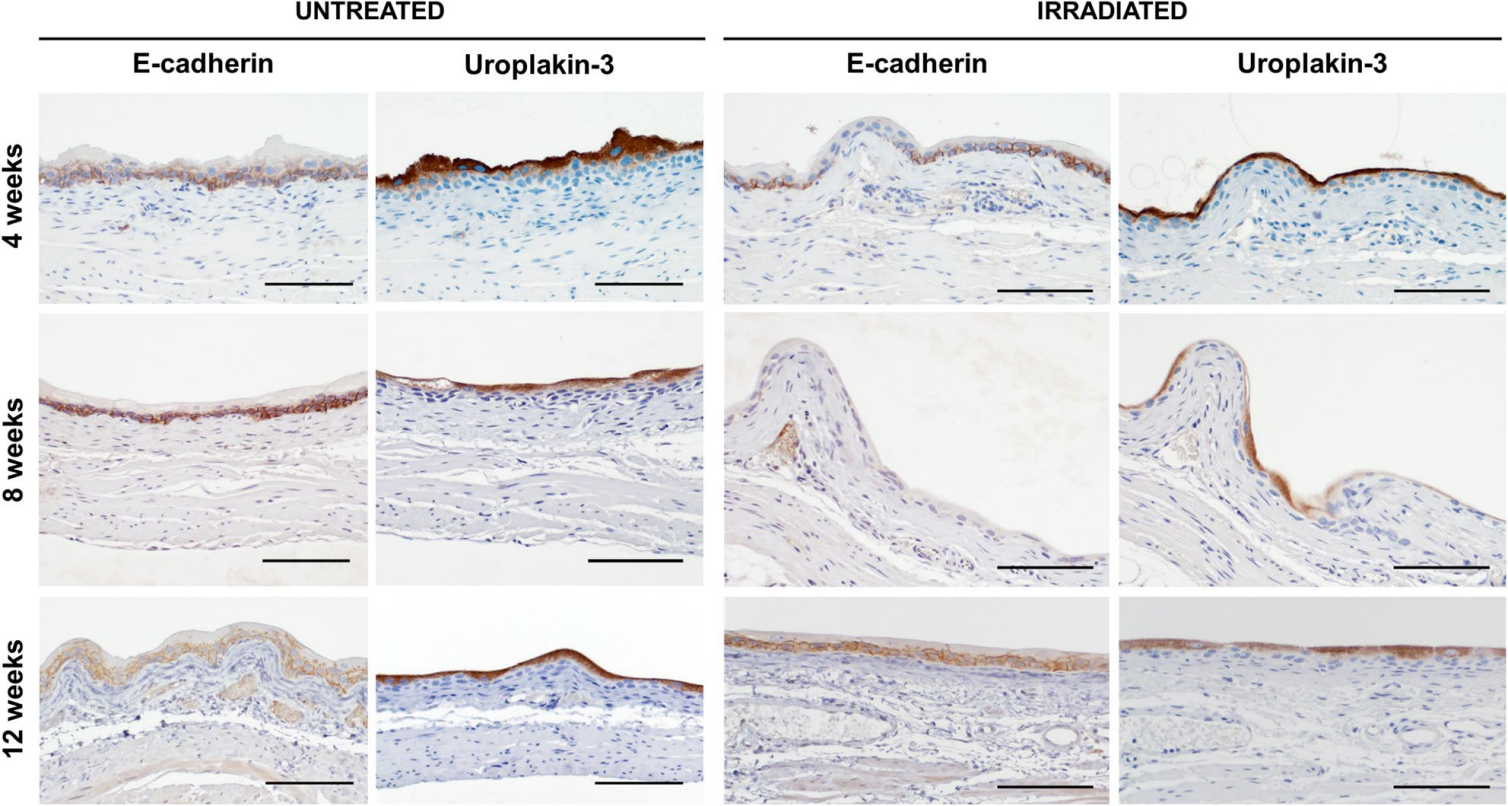
Loss of urothelial cell-adhesion (E-cadherin) and Uroplakin III associated with acute phase of RC. Bladder sections of untreated and irradiation mice at 4-, 8- and 12-weeks post-irradiation were stained with E-cadherin or Uroplakin III (brown) and counterstained with hematoxylin (blue). Scale bar = 100 µm.

**Figure 4:**
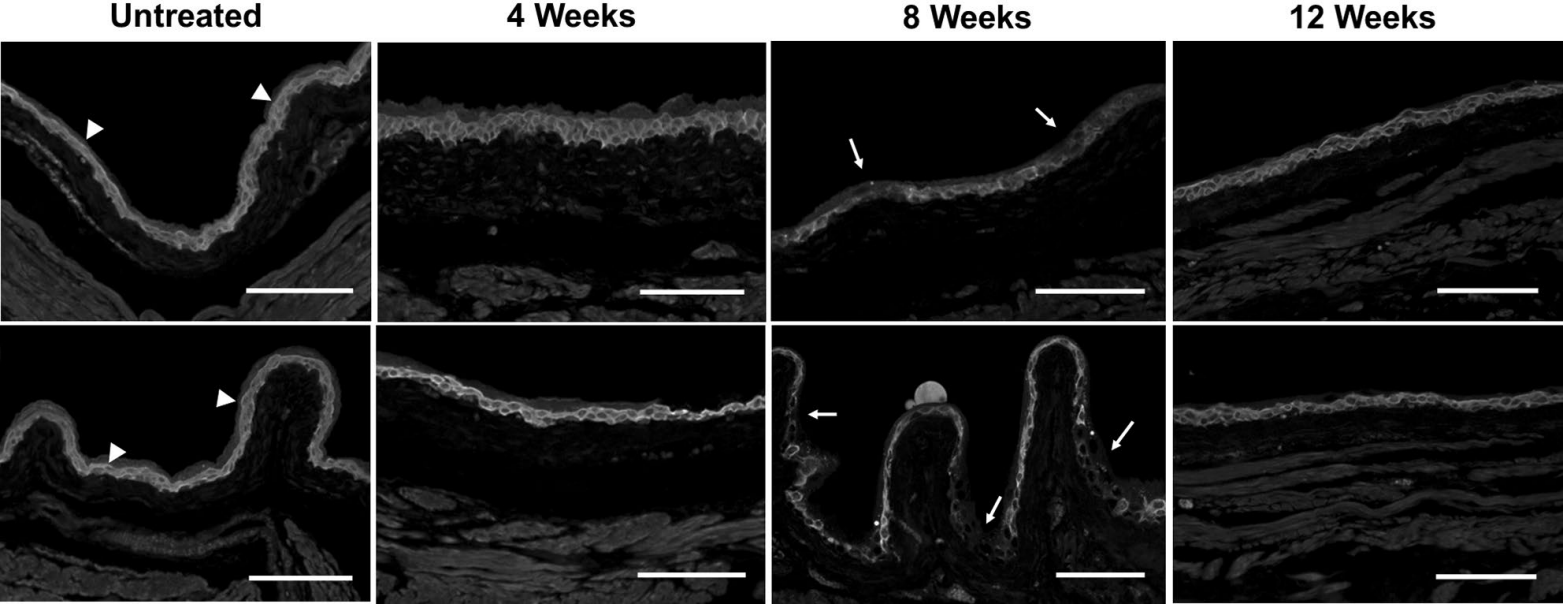
Transient loss of tight junction protein ZO-1 at 8 weeks after irradiation. Bladder sections of untreated and irradiated bladders at 4, 8 and 12 weeks were stained for ZO-1 using immunofluorescence. Arrow heads: positive staining for ZO-1 in the urothelium; Arrows: areas of negative staining for ZO-1. Scale bar = 100 µm.

Bladder sections of irradiated and control bladders were stained with H&E and analyzed for visible histological changes to the bladder. There was a main focus on inflammation, fibrosis and urothelial integrity as these have been shown to attenuate bladder function in other benign bladder conditions such as interstitial cystitis^15^. We first sought to investigate if altered urothelial integrity was a possible mechanism for the observed bladder dysfunction. Bladder sections stained with H&E showed thinning of the urothelial wall starting at 4 weeks after irradiation and peaking at 8 weeks (Fig. 2A,C). By 12 weeks post-irradiation, albeit improved from the 8-week time point, the thickness of the bladder urothelium was still significantly thinner in irradiated versus control bladders. To determine if radiation treatment affected bladder permeability, bladder sections were stained for the cell–cell adhesion molecule E-cadherin and for Uroplakin III. Loss of uroplakin III is associated with increased urothelial permeability, while E-cadherin is an essential cell–cell adhesion molecule to maintain epidermal barrier function^16,17^. Uroplakins are essential structural components of the urothelial apical surface and contribute to urothelial permeability barrier function^18^. E-cadherin expression was partially lost in the urothelium at 4 weeks post-irradiation, and was largely gone by 8 weeks after treatment (Fig. 3). By 12 weeks, E-cadherin expression and localization was similar to that seen in control bladders (Fig. 3). Uroplakin III was also lost after radiation treatment, though was preceded by loss of E-cadherin (Fig. 3). By 12 weeks post-radiation exposure, Uroplakin III levels were gradually increasing (Fig. 3). Finally, we investigated the expression of ZO-1, a tight junction protein, in the bladder urothelium and identified a loss of ZO-1 at 8 weeks after irradiation (Fig. 4). This loss was seen by a decrease in intensity as well as spotty loss of ZO-1 within the urothelium. At 12 weeks post-irradiation, ZO-1 expression pattern and levels were similar to untreated.

### Active inflammation and fibrosis not detected during acute phase of RCs

**Figure 5:**
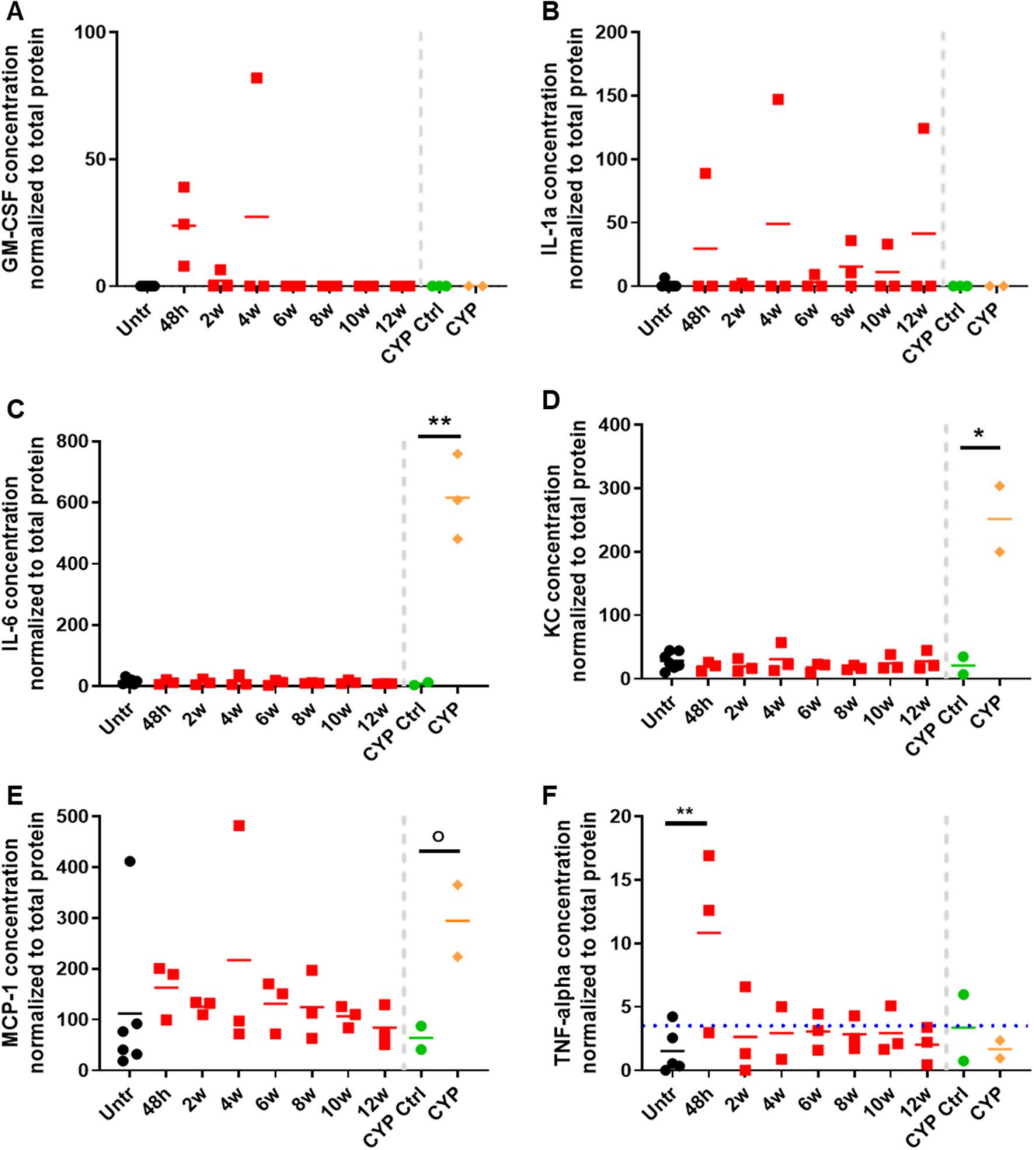
Levels of inflammatory cytokines are unaltered during the acute phase of RC. Cytokine levels were measured in whole bladder lysates from untreated (n = 3) and irradiated (n = 3/time point) bladders. Cyclophosphamide (n = 3) treated bladders and littermate controls (n = 3; CYP ctrl) were included as positive control for inflammation. *GM-CSF* granulocyte macrophage colony stimulating factor, *IL* interleukin, *KC* keratinocyte-derived cytokine, *MCP-1* monocyte chemoattractant protein-1, *TNF-alpha* tumor necrosis factor-alpha; Blue dotted line: lowest range of assay detection.

In addition to compromised urothelial integrity, inflammation and fibrosis have been shown to alter bladder function^19,20^. We investigated the presence of inflammation through histology and protein analysis. As a control for inflammation and tissue damage, histology was compared to bladders from mice that had received CYP-treatment, which has been demonstrated to induce potent and acute inflammation in the bladder^21^. Bladder inflammation, as indicated by an influx of inflammatory cells or edema, was not detected at 4, 8, or 12 weeks after irradiation (Fig. 2A), though we did detect edema in bladders of mice treated with CYP (Fig. 2B). CYP treatment also resulted in thinning of the urothelium and hemorrhaging (Fig. 2B). To determine if radiation resulted in an inflammatory response, we performed multiplex Luminex analysis for a panel of pro-inflammatory cytokines on frozen bladder tissues with and without radiation treatment (Fig. 5). As expected, CYP treatment significantly enhanced the expression of Interleukin-6 (IL-6) and keratinocyte-derived cytokine (KC/CXCL1). No change in pro-inflammatory cytokines was detected in response to irradiation, except for TNF-alpha at 48 h after irradiation. However, TNF-alpha levels had returned to baseline by the 2-week time point.

**Figure 6:**
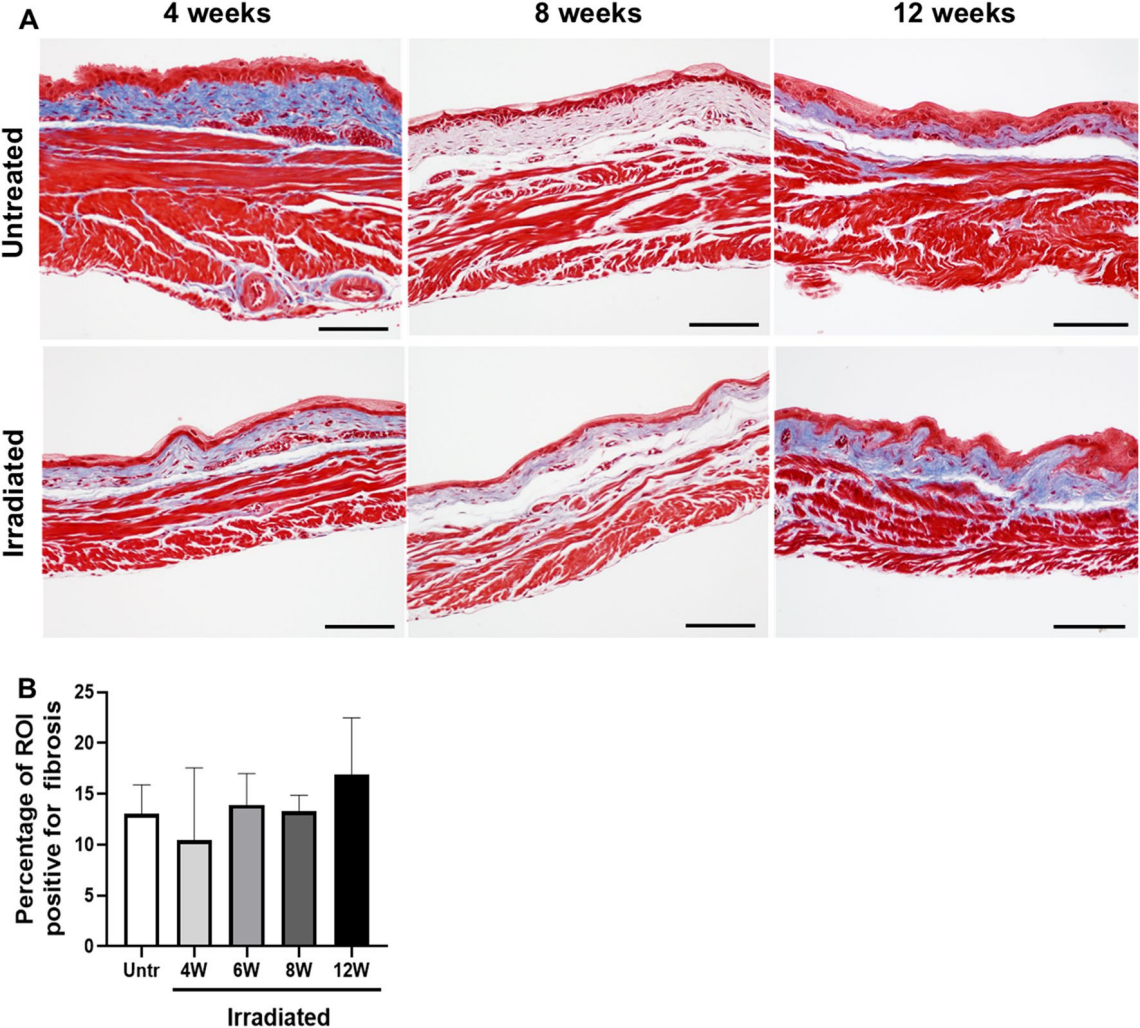
Amount of fibrous tissue in the bladder is unaltered during the acute phase of RC. (**A**) Masson’s trichrome staining was performed on untreated and irradiated bladder sections at 4, 8, and 12 weeks after radiation treatment. (**B**) Quantification of fibrous tissue was performed for untreated (n = 3) and irradiated (n = 3/group) bladder tissues. Fibrous tissue = blue; Scale bar = 100 µm.

In addition to inflammation, we determined the amount of fibrosis in the bladder. Bladder sections were stained using Masson’s Trichrome stain (Fig. 6A) and quantified using ImageJ software (Fig. 6B). Using this method, no increase in fibrosis was detected in bladders up to 12 weeks after radiation treatment. Thus, our study did not find any evidence of inflammation or fibrosis in the bladder in the first 12 weeks after radiation exposure.

## Discussion

Radiation therapy is an efficient method to kill cancer cells. In doing so, damage to normal cells is inevitable. Normal tissues are damaged by radiation through direct exposure or indirectly through the “bystander effect”, through cell signaling between exposed and unexposed cells. The radiosensitivity of cells and tissues is dependent on external factors, such as treatment design (e.g. radiation dose, fractionation), and internal factors, such as genetics and the biological role of the exposed cell. The proliferation rate of cells within a tissue is an important determinant of a tissues radiosensitivity. Cells that are in a resting stage (G_0_) in the cell cycle are relatively radioresistant, as are cells that are in the S-phase because of high levels of enzymes and proteins responsible for accurate DNA replication and repair. On the contrary, cells undergoing mitosis are highly sensitive to radiation damage due to high risk for DNA double strand breaks and lack of repair mechanisms. Overall, the main cell types, e.g. urothelium and detrusor, of the bladder have a low cell-turnover rate: The basal urothelial cells have a turnover rate of 3–6 months^18,22^. The cell-turnover rate of muscle cells can be as high as 30% annually in skeletal muscle to as little as 0.3% annually in adult cardiomyocytes^23,24^. Thus, the bladder is considered an organ with low radiosensitivity, which could help explain the long latency of chronic radiation cystitis which we have previously shown to be mainly driven by vascular damage, loss of muscle tissue and accumulation of fibrosis^10^.

Despite the slow turnover of bladder cells, radiation treatment does induce an acute transient bladder phenotype as observed by increased voiding frequency and decreased void volume 4–8 weeks after irradiation treatment. We observed that bladders exposed to radiation treatment had thinning of the urothelium starting at 4 weeks post-irradiation which became most pronounced at 8 weeks after treatment. By 12 weeks, at which voiding defects were no longer detected either the urothelium had not fully returned to normal thickness. In conjunction with thinning of the urothelium, irradiation induced loss of the cell–cell adhesion protein E-cadherin, which was restored by 12 weeks post-irradiation. At 4 weeks post-irradiation, loss of E-cadherin is spotty throughout the bladder and by 8 weeks E-cadherin is lost throughout most of the urothelium. A urothelial defect does not need to be uniform throughout the bladder to cause significant symptoms. Heterogeneity is common in other conditions, such as interstitial cystitis where bladder pain/symptoms can be caused by only one or several lesions within the bladder. In addition, E-cadherin needs to be localized to the cell membrane for it to exert its cell–cell adhesion role. A similar mechanism has been described for radiation-induced acute dermatitis, where a high influx of reactive oxygen species induces loss of E-cadherin resulting in a destruction of the epidermis^25^. Loss of E-cadherin has been identified as an early trigger of cell apoptosis in response to photodynamic treatment, and^26^. In contrast to our findings, a recent study by Krischak and colleagues demonstrated that bladder irradiation resulted in an increase in E-cadherin expression between 3 and 30 days post-irradiation when compared to non-treated littermate controls. The difference in study findings could be explained by the lower radiation dose used by Krischak and colleagues (23 Gy versus 40 Gy). In addition Krischak and colleagues used C3H/neu mice, which we have previously shown to be more resistant to bladder radiation damage than C57BL/6 mice as measured by amount of fibrosis within the bladder^10^. However, the contradicting findings between C3H/neu and C57BL/6 mouse strains does suggest that certain patients might have an inherent genetic predisposition to developing acute RC symptoms.

In addition to loss of E-cadherin, we identified loss of Uroplakin III after irradiation. Decrease in Uroplakin III was secondary to E-cadherin loss, suggesting that loss of Uroplakin III was a side-effect from loss of urothelial cells. Uroplakin III interacts with other Uroplakin proteins to form plaques on the apical surface of the urothelium. Here it contributes to the impermeable nature of the bladder. Previous studies have shown that ablation of Uroplakin III is sufficient to disrupt these urothelial plaques and cause urothelial leakage, which subsequently results in voiding dysfunctions^27,28^. Thus, the loss of Uroplakin III after irradiation is suggestive of a leaky bladder phenotype that can induce the observed voiding dysfunction.

In conjunction with the loss of E-cadherin, the tight junction protein ZO-1 was also reduced and lost at varying places throughout the urothelium at the 8-week timepoint. Tight junction proteins such as ZO-1 play an important role in maintaining the urothelial barrier. In fact, dysregulation of the tight junction barrier has been shown to sensitize the bladder urothelium, and loss of ZO-1 has been reported in the urothelium of patients with Interstitial Cystitis^29,30^. Thus, the loss of ZO-1 in bladder urothelium after irradiation supports the hypothesis that irradiation induces urothelial dysfunction that may induce the associated voiding defect.

Finally, we investigated a possible involvement of inflammation or fibrosis to the observed voiding defect. Voiding defects such as urinary frequency and decreased void volume are common in patients suffering from urinary tract infections or interstitial cystitis, as well as patients with a fibrotic bladder^20,31,32^. Based on histology and protein analysis, we did not observe any significant increase in pro-inflammatory cytokines, while we did see an increase in IL-6, MCP-1 and KC in cyclophosphamide treated bladders. Cyclophosphamide induces an immediate and significant inflammatory response in the bladder. It has historically been used as a preclinical model for interstitial cystitis and was used here as a positive control for inflammation. It is feasible that other cytokines or chemokines are elevated, but we were limited to the analytes available for the assay. A significant increase in TNF-alpha was detected in irradiated samples at 48 h after radiation exposure. TNF-alpha acts as an inflammatory mediator by triggering the expression of cytokines and chemokines including IL-1, IL-6, and MCP-1. However, we failed to detect an increase in either cytokine, thus the increase in TNF-alpha might not be significant enough to induce an inflammatory response^33^. TNF-alpha plays a role in many other cellular processes such as cell death, cell adhesion, cell proliferation and extracellular matrix remodeling, thus the acute elevation of TNF-alpha could be impacting other cellular processes^33^. In urine samples from human prostate cancer survivors with history of pelvic radiation therapy, we also failed to detect inflammatory cytokines such as TNF-alpha, though these urine samples were taken from patients beyond the acute phase of RC^11^. In this study we were also unsuccessful at detecting signs of fibrosis at the time micturition defects were observed, suggesting that accumulation in extracellular matrix did not contribute to the observed bladder dysfunction. We have previously shown that fibrosis is a major pathological feature in chronic RC and thus must become apparent at a later stage in the disease progression.

One limitation of the study is the small group sizes. It is feasible that repeating the study with a larger n-value per time point would yield different results. For example, a significant increase in certain cytokines 48 h after irradiation, such as GM-CSF, might become apparent with an increase in study subjects. However, in our experience using this preclinical model of radiation cystitis, we have thus far not detected any histological signs of inflammation^10,13^.

In summary, using our preclinical model of RC, we show that irradiation results in an increase in micturition frequency and decrease in average void volume 4–8 weeks after treatment, and this voiding defect coincided with a significantly compromised urothelial integrity as measured by loss of urothelial cells, E-cadherin, ZO-1 and uroplakin III. We hypothesize that symptoms of acute RC are caused by damage to the urothelium, while symptoms during chronic RC are the result of fibrosis and vascular damage. This may allow the clinicians an opportunity for early interventions for patients suffering from acute RC by treating or preventing the urothelial barrier defect. Our model of RC and observations in both the acute and chronic phase may help toward developing treatments for and methods to prevent long term complications and enhance patient quality of life in cancer survivors after pelvic radiation.

## Supplementary Information

Supplementary Information.

## Data Availability

All data generated or analysed during this study are included in this published article (and its Supplementary Information files).
