# Supplementary material for: Voiding defects in acute radiation cystitis driven by urothelial barrier defect through loss of E-cadherin, ZO-1 and Uroplakin III
Source: Sci Rep. 2021 Sep 29;11:19277. doi: 10.1038/s41598-021-98303-2 (PMC8481534; doi:10.1038/s41598-021-98303-2)
Supplement: Supplementary file 1 — Supplementary Information. [file 41598_2021_98303_MOESM1_ESM.pdf]

Supplemental Information

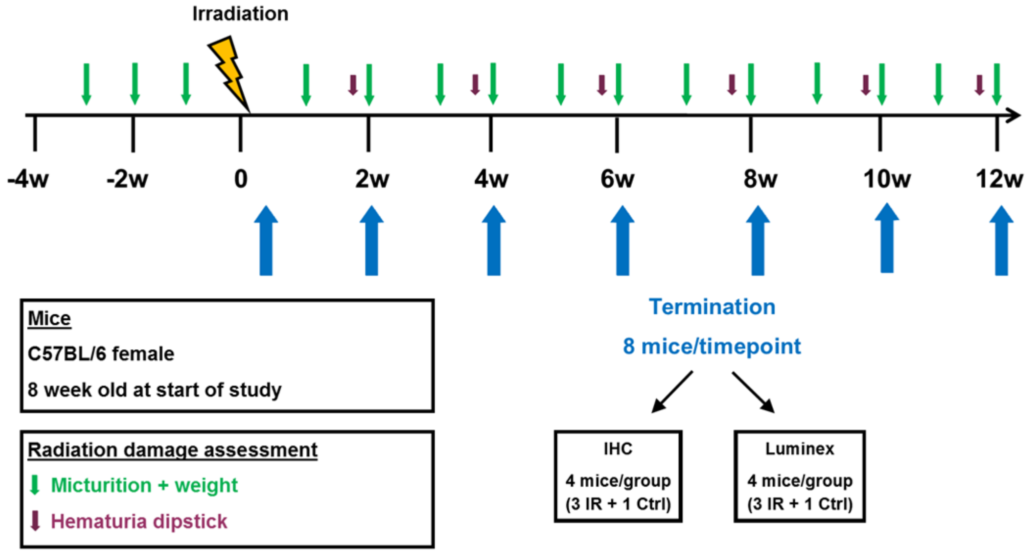

Supplemental Fig 1: Experimental study design. Irradiation was performed at time zero. Mice underwent weekly micturition assessment starting at 2 weeks prior to irradiation. Prior to sacrifice, urine dipstick analysis was performed for hematuria. Eight mice were sacrificed at the indicated time points (biweekly) and bladder tissues harvested. Four bladders (3 irradiated and 1 control) were processed for histology, and the remaining bladders were processed for protein analysis.

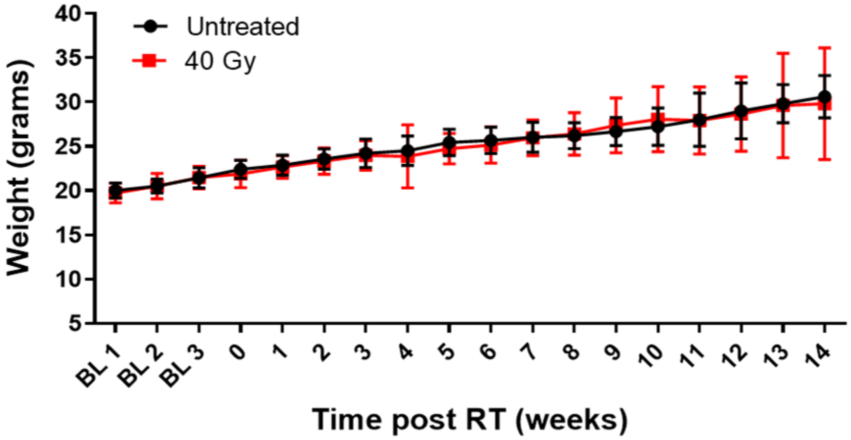

Supplemental Fig 2: Mice maintained normal body weight after radiation treatment. Mice were weighed weekly after radiation treatment to monitor overall health. Black circle: untreated; Red square: 40 Gy. Error bars = SD.
